# Cerebro Arachnitis

**Published:** 1852-01

**Authors:** J. B. Nash

**Affiliations:** Dixon, Ill.


### ARTICLE IV.

*Cerebro-Arachnitis.* By J. B. NASH, M. D., of Dixon, Ill.

I noticed in the November number of the North-Western Medical and Surgical Journal an article on cerebro-arachnitis, which was read before the Central Medical Society, of Illinois, by Dr. James Smick.

I was much pleased with the able manner in which he described the disease. It corresponded almost exactly with the disease as it has prevailed in this vicinity. But as I have been pursuing a somewhat different course from that described by Dr. S. and other physicians who have written upon the disease, I will give an account of my treatment and its results.

I will say nothing about the symptoms as Dr. S. has given them as correctly and much more ably than I could.

The disease broke out here in the winter of 1840, and assumed a very malignant form. The first case I was called to see I found in articulo mortis twenty-four hours after the attack. The next two cases I saw twelve hours after they were attacked; I bled, used external applications, gave cathartics, but could get no operation from them. They died in nine and twelve hours. In company with Drs. Everett and Tiedman we made post-mortem examinations in the last two cases. We found unequivocal signs of congestion of the brain and inflammation of its meninges.

The course I then adopted was to bring about re-action in the first place, and after it was established, to use the most vigorous antiphlogistic treatment, by which course I saved the most of my patients.

In 1841, the disease again made its appearance. In the first case I could only bring on a partial re-action. The patient soon died. The next case was a little girl. She was attacked with the usual symptoms. Her stomach was so irritable that she could keep no medicine upon it. I was afraid to give an emetic, as I had been taught that in all diseases of the brain and determinations of blood to the head, emetics were inadmissable. I, however, ventured to give a light one of ipecac, which operated gently, relieving the congestion of the brain and irritation of the stomach. I then enquired whether it was a fact or not, that emetics were inadmissable in determinations of blood to the head, and concluded they might be, when inflammation had actually taken place, but previous to that, or while congestion only existed, I thought they might prove valuable by equalizing the circulation. In my next case I gave a strong antimonial emetic, which at once relieved the patient, a gentle laxative the ensuing day completing the treatment. After this I treated about thirty cases during the winter, and have had the care of more or less of the disease almost every year since, which I have treated upon the same plan. I have not lost any cases during this time that I have seen before inflammation had taken place.

In some cases of strong plethoric persons, I have bled previously to giving the emetic, if re-action was fully established before I saw them. In some, after giving the emetic, I have found it necessary to give an active cathartic, (such as a dose of calomel followed with salts and senna,) and sometimes I have used diaphoretics for a day or two.

If on the next day or the day after I discover any symptoms of another exacerbation (which is always light after the above treatment,) I give quinine.

I never wait for re-action to take place before giving the emetic, but the sooner it is given, after the attack, the better; as I have found nothing that so speedily relieves congestion and restores the circulation to its normal standard.

I always leave an opiate to be given, if the emetic acts upon the bowels, for fear the antimony might produce intestinal irritation which invariably retards the recovery of the patient.

